# Ubiquitin recruiting chimera: more than just a PROTAC

**DOI:** 10.1186/s13062-024-00497-8

**Published:** 2024-07-09

**Authors:** Tatyana A. Grigoreva, Daria S. Novikova, Gerry Melino, Nick A. Barlev, Vyacheslav G. Tribulovich

**Affiliations:** 1 Laboratory of Molecular Pharmacology, St. Petersburg State Institute of Technology (Technical University), St. Petersburg, 190013 Russia; 2 Department of Experimental Medicine, University of Rome Tor Vergata, Rome, 00133 Italy; 3 Institute of Cytology RAS, Saint-Petersburg, 194064 Russia; 4 Department of Biomedical Studies, School of Medicine, Nazarbayev University, Astana, 010000 Kazakhstan

**Keywords:** Ubiquitin, PROTAC, Protein degradation

## Abstract

Ubiquitinylation of protein substrates results in various but distinct biological consequences, among which ubiquitin-mediated degradation is most well studied for its therapeutic application. Accordingly, artificially targeted ubiquitin-dependent degradation of various proteins has evolved into the therapeutically relevant PROTAC technology. This tethered ubiquitinylation of various targets coupled with a broad assortment of modifying E3 ubiquitin ligases has been made possible by rational design of bi-specific chimeric molecules that bring these proteins in proximity. However, forced ubiquitinylation inflicted by the binary warheads of a chimeric PROTAC molecule should not necessarily result in protein degradation but can be used to modulate other cellular functions. In this respect it should be noted that the ubiquitinylation of a diverse set of proteins is known to control their transport, transcriptional activity, and protein-protein interactions. This review provides examples of potential PROTAC usage based on non-degradable ubiquitinylation.

## Background

The ubiquitin recruiting small molecule was first introduced in 2001, when it was shown that the chimeric compound Protac-1 is capable of triggering ubiquitinylation of a protein target, leading to its proteolysis [1]. This technology was dubbed as PROTAC (PROteolysis TArgeting Chimera) and became the first in a series of PROTAC-like techniques that artificially forced post-translational modifications (ubiquitinylation, phosphorylation, and acetylation) of the target proteins due to the chemically-arranged physical proximity to the corresponding enzyme. The concept of induced protein interaction has been confirmed in recent years by a number of successful large-scale studies. In particular, 17 drugs based on protein proteolysis provoked by ubiquitinylation are undergoing clinical trials by 2023 [2].

**Fig. 1:**
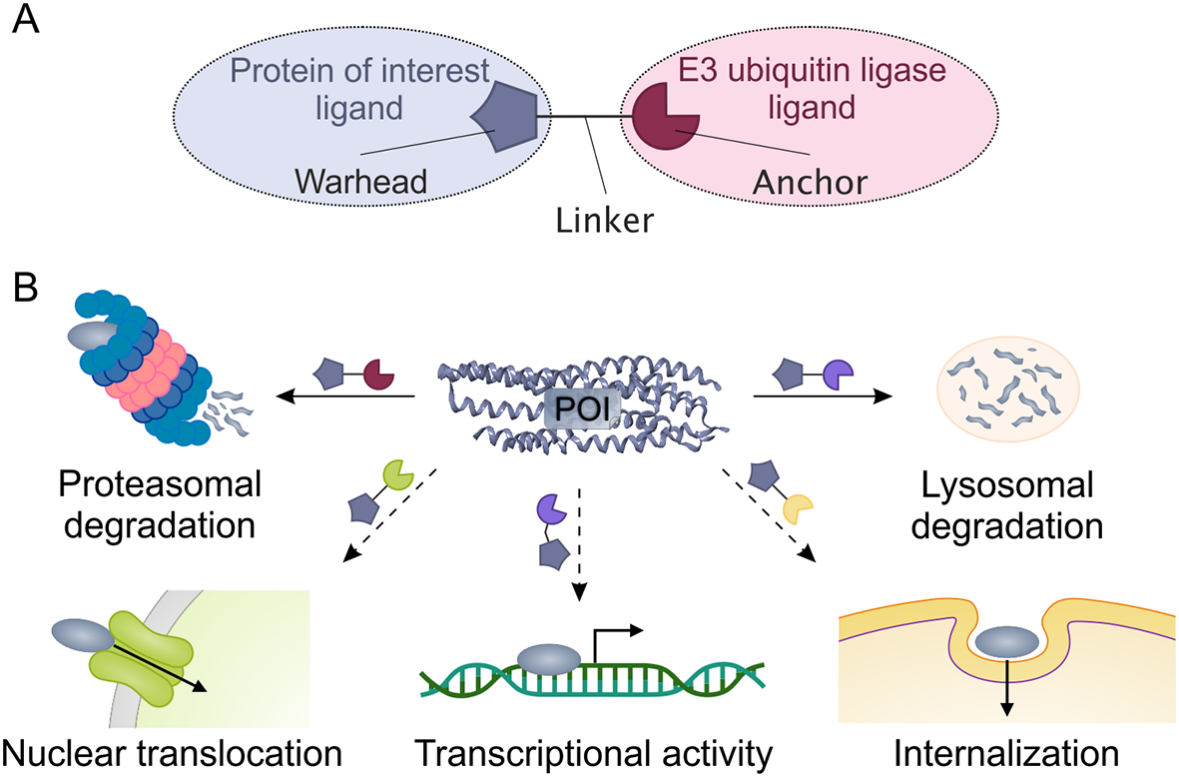
PROTAC principle (**A**): chimeric molecule brings a protein of interest and an E3 ubiquitin ligase together upon binding of its warhead to the protein of interest and its anchor to the E3 ubiquitin ligase, while the warhead and the anchor are connected by the linker of a certain length providing further ubiquitinylation. Prospects for the use of ubiquitin recruiting chimera (**B**): while the direction of ubiquitin-mediated proteolysis (PROTAC technology) is actively developing, other modalities of forced selective ubiquitinylation of protein substrates, such as modulating transcriptional activity or transport between cellular compartments, remain unexplored

Capitalizing on the wealth of experimental data that has been accumulated over the last two decades of intense research, the PROTAC approach turned into a routine search of the optimal target – linker – ubiquitin ligase combination (Fig. 1A). The choice of elements in the scheme is determined not so much by the pharmacological and/or research suitability, but by the availability of the relevant tethering compounds. The latter is reflected by the impressive growth of commercially available building blocks for the construction of binary warheads molecules. However, in parallel, data on the involvement of ubiquitin not only in the proteasomal degradation of proteins, but also in other cellular processes were revealed [3–7]. All this opens up promising prospects for a significant expansion of the application range for ubiquitin-targeting chimeras.

Ubiquitinylation is involved in regulation of the wide range of cellular processes and is not limited to the protein degradation. Thus, manipulation of ubiquitinylation by fine-tuning the site of ubiquitinylation and the specificity of the ubiquitin chain allows modulating the protein conformation as well as its interactions with other macromolecules including transcriptional activity, the protein transfer between organelles, selective blockage of different stages of cell cycle and others (Fig. 1B). The desired effect can be achieved through careful selection of the executor E3 ligase, many of which are yet unexplored, and the development of new small molecule ligands that can bind them.

The present review highlights the directions in which one can develop rational design of PROTAC based on forced ubiquitinylation, since such modification of proteins can provoke not only commonly expected proteolysis of the target, but also cause diverse, but predictable consequences for a specific target.

## PROTAC

The concept of PROTAC was developed by Deshaies group in 2001 [1], and soon after it gained wide popularity in the field of molecular oncology because it proved to be very useful for the functional analysis of cancer-related kinases and their substrates.

Such a molecular engineering approach using bifunctional chemical probes looks like a versatile tool for targeted degradation of specific proteins. These bifunctional chemical probes consist of two different small molecules linked together where one of them binds the target protein and the other one recruits an E3 ligase, an enzyme that covalently modifies proteins destined for degradation in the proteasome (Fig. 1A) [8].

**Fig. 2:**
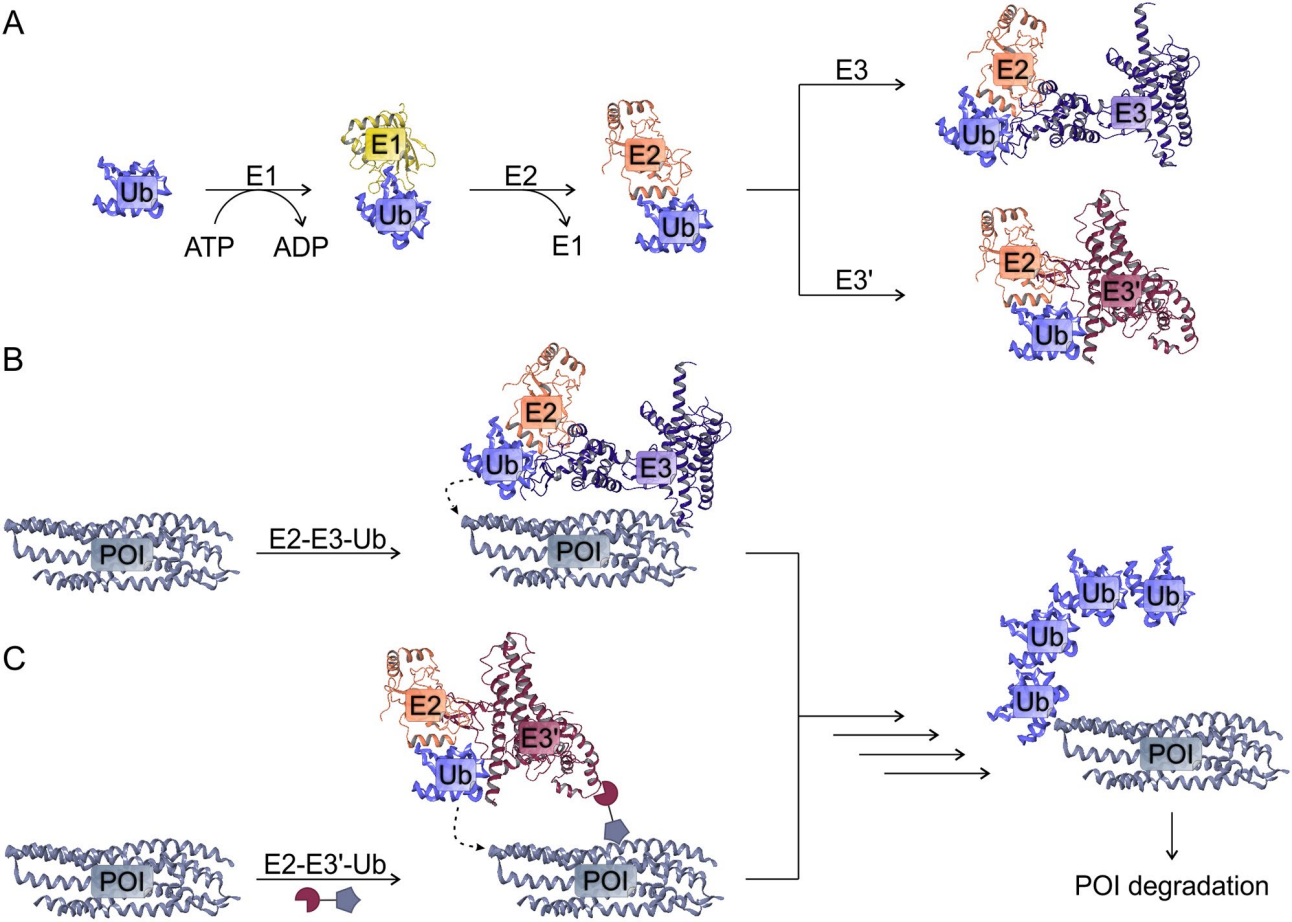
PROTAC in proteasomal degradation. Ubiquitin activation and formation of ubiquitinylation complex (**A**): the ubiquitin molecule is activated by the formation of a high energy bond between the residue Gly76 of ubiquitin and the cysteine residue of E1; then E1 attaches the activated Ub to E2 providing the binding platform for E1, E3, and activated Ub. Natural ubiquitinylation of the target by E3 ligase (**B**): highly selective E3 ensures specific recognition of a specific target protein. Ubiquitinylation of the target by PROTAC (**C**): PROTAC ensures recruitment the desired E3 to the target protein

E3 ligase is an enzyme in the cascade ensuring the attachment of a specific tag, ubiquitin, to proteins, which acts as a signal for protein breakdown in the proteasome. In general, this cascade consists of 4 stages, where ubiquitin is sequentially transferred between several enzymes (E1-E2-E3) until it is finally covalently attached on the target lysine of the substrate protein (Fig. 2). Firstly, the ubiquitin molecule is activated due to the ATP-dependent formation of a high energy thioester bond between the Gly76 residue of ubiquitin and the cysteine residue of E1; then E1 connects the activated Ub to E2, which provides a binding platform for E1, E3, and activated Ub. The participation of highly selective E3 ensures specific recognition of a specific target protein, to the lysine of which several ubiquitins are sequentially attached to form a polyubiquitin chain. Lysine is an abundant amino acid (6% of the entire proteome) and its ε-amine mediates many protein-protein interactions [9], accordingly, each protein has several potential ubiquitinylation sites [10]. Conjugation of multiple ubiquitin units to monoubiquitylated substrates is conducted by E4 ubiquitin-chain elongation factors or E3 ligases with such an activity [11]. The buildup of the polypeptide tag provokes the final stage of the cascade, protein cleavage by the 26S proteasome [12]. In this case, polyubiquitin chains are successfully recognized by proteasomal ubiquitin receptors Rpn1, Rpn10, and Rpn13 of the 19S sub-complex, which initiates protein translocation to the 20S sub-complex for subsequent degradation [13]. Importantly, enzymatic activities of proteasomes may be regulated by ubiquitinylation [14, 15]. Several groups including ourselves have shown that various forms of cellular stress control the activity of 20S proteasomes via post-translational modifications [16, 17].

Although more than 600 E3 ligases are known, which vary significantly both in structure and physiological functions [18], only four ubiquitin ligases are used for the PROTAC-mediated approach: VHL, MDM2, IAPs (inhibitor of apoptosis proteins), and CRBN (cereblon). The major bottleneck for the wider use of the wealth of different E3 ligases in the PROTAC system is the lack of specific chemical probes that would ensure their specific and robust tethering to their respective targets. The latter underscores the necessity of structure-function studies and chemical screening of various libraries to identify additional E3 ligase-targeting probes.

**Fig. 3:**
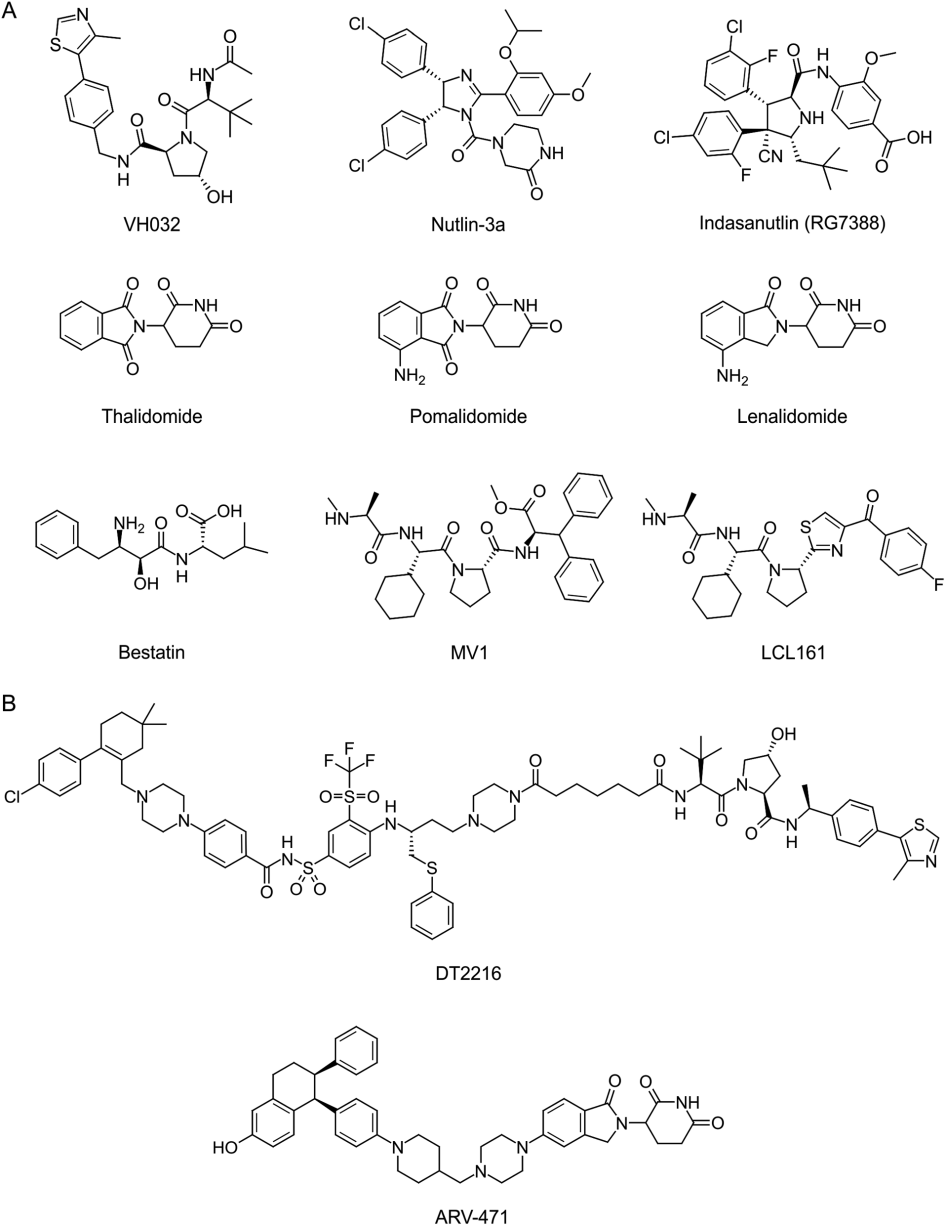
Structures underlying the most common PROTACs: VHL inhibitors, MDM2 inhibitors, CRBN ligands, IAP antagonists (**A**); examples of PROTACs in clinical trials (**B**)

The von Hippel–Lindau (VHL) tumor suppressor substrate receptor interacts with the CRL2 complex (adaptor subunits Elongin B and Elongin C, scaffold subunit Cullin 2, and the RING-containing protein Rbx1), forming Cullin RING ligase complex CRL2^VHL^ [19, 20]. In 2014, VH032, an inhibitor of the VHL E3 ligase interaction with the hypoxia inducible factor HIF-1α, was proposed [21], which formed the basis of VHL-binding PROTACs (Fig. 3A) [22]. In 2024, stage I clinical trials of BCL-X_L_-targeted PROTAC DT2216 (Fig. 3B) in patients with relapsed/refractory malignancies was completed [23].

In the case of the MDM2 E3 ubiquitin ligase playing a key role in the regulation of apoptosis, a number of inhibitors of various chemical classes have been developed to date [24–26]. The pharmacophore concept of MDM2 inhibitors was developed allowing the rational design of the strength of chimeric molecule binding to the enzyme substrate [27–29]. Such PROTACs are currently constructed based on the nutlin series such as nutlin-3a and idasanutlin (Fig. 3A) [30, 31]. It is worth noting that a number of PROTACs consider MDM2 not as an E3 ligase, but a protein of interest (POI) [32]. This also fits into a cost-effective approach based on the use of available tools, i.e. proteins for which there are known small molecule ligands, rather than on the search for new tools to solve new tasks.

Cereblon (CRBN) acts as a substrate-specific receptor in the E3 ubiquitin ligase CUL4-RBX1-DDB1-CRBN(CRL4^CRBN^) complex [33, 34]. It turned out that it is the interaction with CRBN that determines the anti-multiple myeloma activity of thalidomide and analogs [35, 36]. These ligands are readily synthesized, making the design of various CRBN-binding PROTACs relatively simple (Fig. 3A). Similar structures were proposed as degraders of proteins involved in various diseases, including tumors, immune diseases, and neurodegenerative diseases [34, 37–39], and dominate clinical trials; in particular, ARV-471 is undergoing stage III clinical trials [40] against advanced-stage ER^+^HER2^−^ breast cancer (Fig. 3B).

IAP (inhibitor of apoptosis proteins, such as cIAP1 and XIAP) antagonists, which possess ubiquitin ligase activity, are also used to label protein targets, and it is assumed that their concomitant autoubiquitinylation may contribute to the therapeutic effect [41, 42]. Among the ligands similar to specific and nongenetic IAP-dependent protein erasers (SNIPERs) are bestatin, MV1, LCL (Fig. 3A).

As it has been mentioned earlier, the choice of ligase at the moment is determined not so much by rational analysis of potential effects, but by the availability of well-known and accessible ligands for synthesis. It is this factor that unites the above mentioned enzymes which provide proteolysis of their targets.

On the other end, the only condition for proteins that are targeted by the PROTAC approach is the availability of small molecule ligands capable of potent and selective binding to them [43, 44]. For example, PROTACs for focal adhesion kinase based on the FAK inhibitors defactinib and PND-1186 [45, 46], BCL-XL targeting PROTACs based on A-1,155,463 (selective inhibitor) [47] and navitoclax (ABT263, a BCL-2 and BCL-XL dual inhibitor) [48], BRD4 degraders based on JQ1 [49, 50] is just a few from the everlasting list of new potential targets.

Considering the predictability of the elements used, from which PROTACs are assembled, the current approach of combining “known ligand of a known target + known ligand of a known ubiquitin ligase” limits the translational advantages of PROTACs and does not allow us to fully exploit the advantages of recruiting ubiquitin, since its role in the cell is far from limited to PROteolysis (Fig. 1B).

## Ubiquitin

**Fig. 4:**
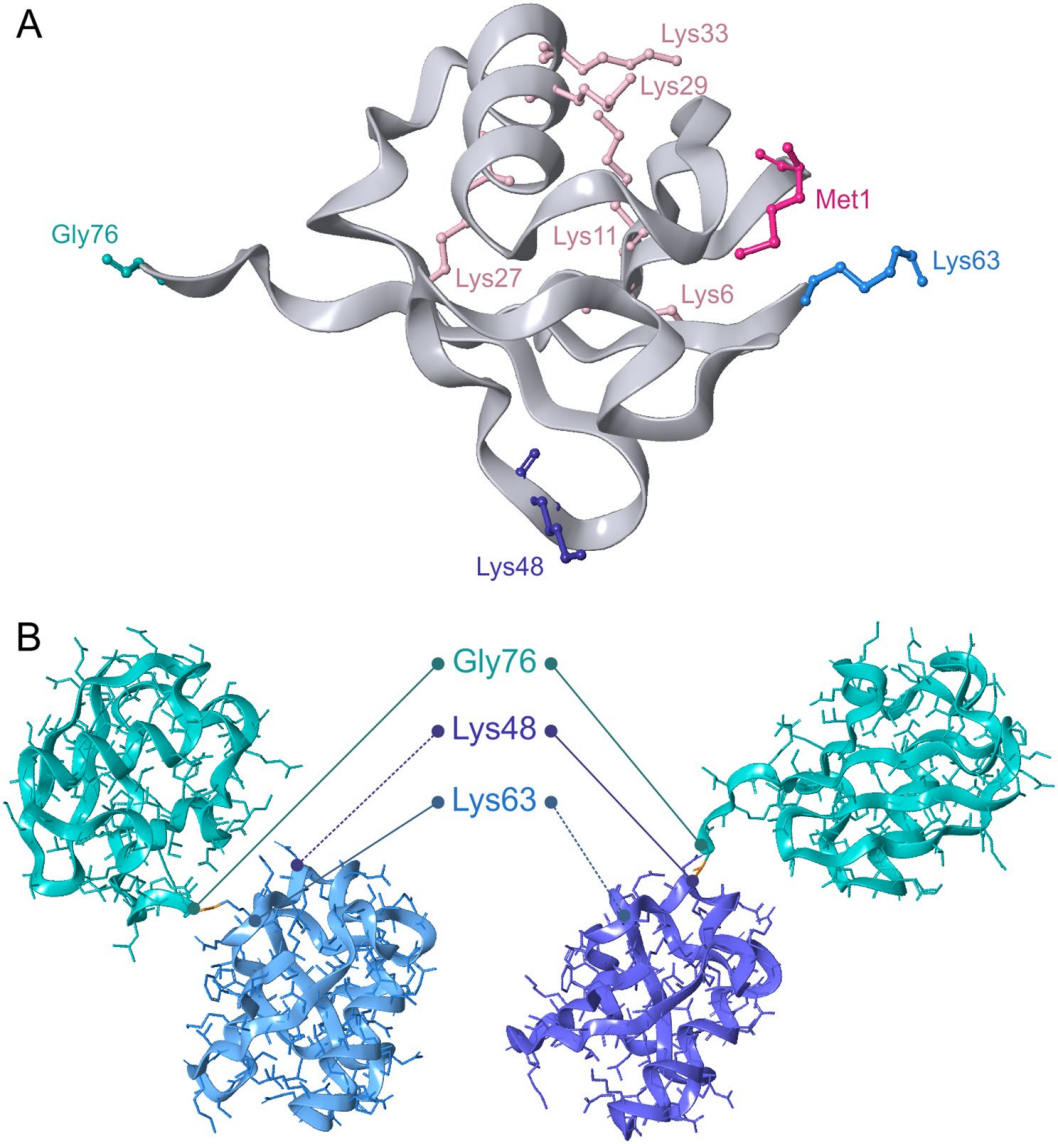
Ubiquitin and its chains. Cartoon representation of ubiquitin; the main residues are shown in colored sticks (**A**). Diubiquitin conjugates: Lys63 linked (left) and Lys48 linked (right) (**B**). PDB IDs used: 3A9K [53] and 6Z7V [54]

Ubiquitin (ubiquitous immunopoietic polypeptide) is a highly conserved compact 8.5 kDa protein; carboxy-terminal tail (glycine) of ubiquitin is exposed, allowing its covalent linkage to target proteins [51]. It is formed by 76 amino acids including 7 lysines (Lys6, Lys11, Lys27, Lys29, Lys33, Lys48, and Lys63), that is, there are 7 potential autoubiquitinylation sites that provide possible formation of various variants of polyubiquitinylation systems (Fig. 4). In addition to lysine residues, ubiquitins can also bind through Met1, forming several similar or different bonds, which allows the formation of polyubiquitin chains of various types: linear and branched, homotypic and heterotypic, as well as to conduct multiple monoubiquitination of targets. Ubiquitin chain initiation, elongation, and branching often requires an intricate cooperation between different E2 and E3 enzymes [52].

The attachment of ubiquitins alters the potential for post-translational modification of the target, potentially competing with SUMOylation, phosphorylation, acetylation, etc. Ubiquitin itself can also undergo various post-translational modifications, such as phosphorylation and acetylation, which in turn affect the charge and surface properties of ubiquitin [6, 55, 56]. Ubiquitins that “tag” the target can interact with a wide range of proteins containing ubiquitin-binding domains (UBDs), including proteasome subunits, which finally results in degradation of the target. Thus, during the regulation of a large number of cellular processes, the multifaceted ubiquitin code is involved, where ubiquitin acts as a signaling component that can trigger molecular events by operating as a reversible and highly versatile regulatory signal for effector proteins – ubiquitin receptors containing one or more ubiquitin-binding domains [51, 57–60].

**Fig. 5:**
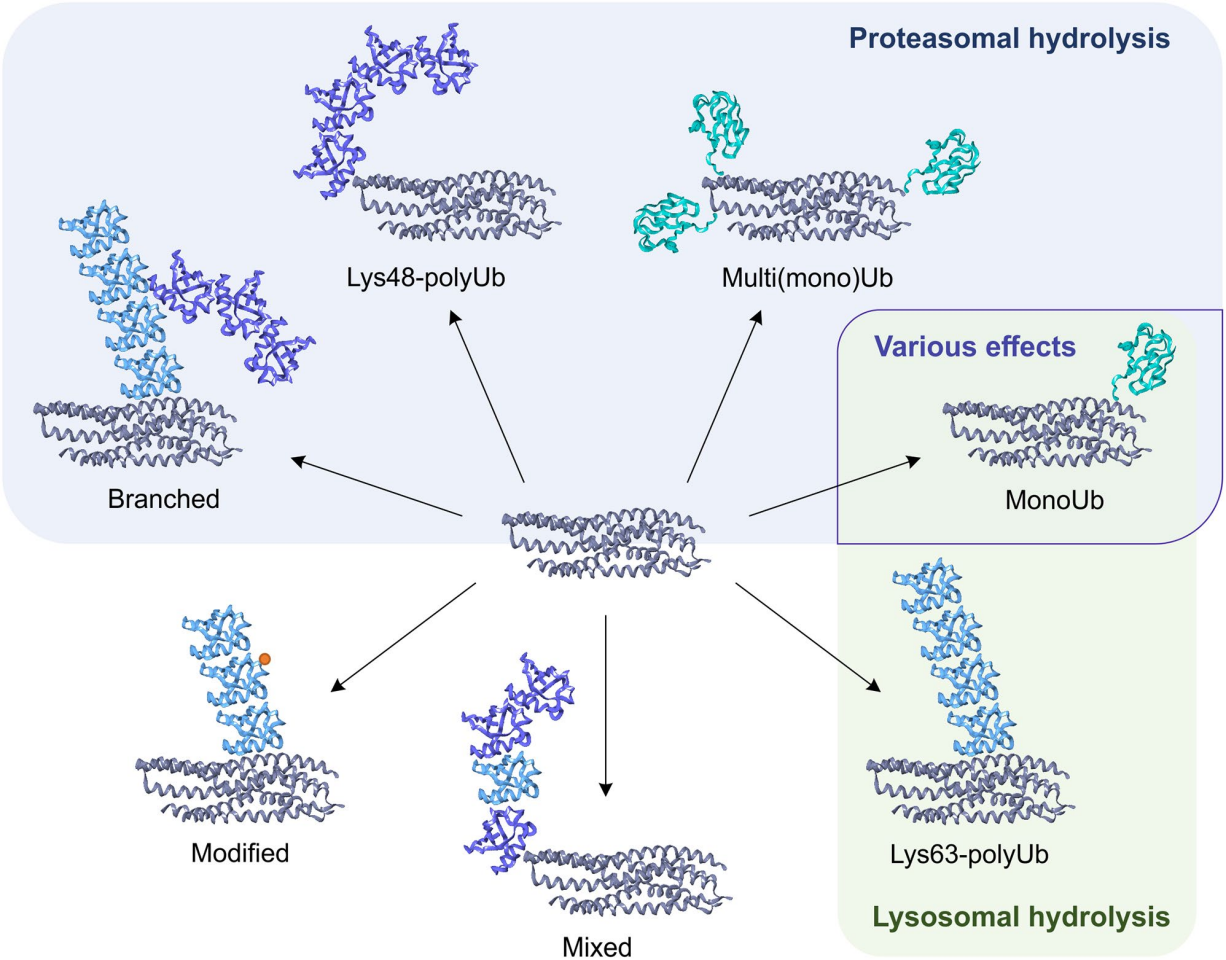
Types of substrate ubiquitinylation and their main effects. Variability in attachment modes allows for the production of diversely configured ubiquitinylated substrates. Differences in the length and composition of the ubiquitin chain allow the substrate to interact with heterogeneous downstream cellular factors leading to various cellular effects

Theoretically, any lysines located on the surface of the target protein can be ubiquitinylated; but in practice this process is strictly regulated and is determined primarily by the specificity of the recruited E3 ligase [10]. On the other hand, many of these enzymes are of low specificity; moreover, mutation of the main ubiquitin site of the target may not interfere with the efficiency of the ubiquitinylation process [61], indicating a more complex regulation than a direct relationship of 1 protein – 1 site – 1 ubiquitin ligase. As a result of the variability in ubiquitinylation, various complexes can be formed (Fig. 5), leading to various cellular effects due to the interaction of the ubiquitinylated substrate with heterogeneous downstream cellular factors, for which both the length of the chain and its branching and modifications are important [10, 62].

### Ubiquitin chains

The ubiquitin code turned out to be very confusing, and has not yet been completely resolved. However, researchers agree that the polyubiquitinylated tail formed by ≥ 4 Lys48 peptide bonds is recognized by the 26S proteasome and, accordingly, provokes hydrolysis of the target protein [10, 63]. Such long ubiquitin tails provide high affinity to the proteasome, promoting target destruction [64]. Perhaps, it could be explained by the fact that the distance between the two best studied ubiquitin receptors of the proteasome, Rpn10 and Rpn13, fits a Lys48-based tetraubiquitin chain [65, 66].

At the same time, chains of similar length formed through Lys63 do not cause a pronounced effect [67]. It is believed that cellular Lys63 chains, which form extended structures with a minimum of intersubunit contacts, have less proteasomal accessibility, and proteasome-bound Lys63 chains are more rapidly deubiquitinated by proteasome elements, which could cause ineffective degradation of Lys63 conjugates. It was noted in [68] that Lys63 polyubiquitin conjugates in cell lysates were rapidly disassembled compared with Lys48 chains. In this case, branched ubiquitin chains of the Lys48/Lys63 composition can act as a substrate-specific mark for proteasomal degradation [69]; the branched chain can be considered as a Lys48 proteasomal tag on the target-Lys63 conjugate. At the same time, a similar branching had the effect of protecting the conjugate from deubiquitinylation in another study, which suggests that branched chains regulate biological pathways via multiple mechanisms and are functionally distinct from mixed or multiple chains [70]. It is worth noting that the Lys63 ubiquitin tail can play the role of a molecular glue that allows for rapid and reversible formation of pivotal signaling complexes [71] and thus involved in many cellular processes, which can also be used to control their activity and direction.

Polymers of other structures can also become a signal for the proteasome [72]. For example, heterotypic Lys11/Lys48 polyubiquitin chains can bind to the proteasome and signal for degradation, although data on the effectiveness of such a signal compared with Lys48 are contradictory [72, 73]. The authors [74] note differences in the use of Lys11 in the ubiquitin code between the proteasomal degradation systems of cytoplasmic and nuclear misfolded proteins. In turn, Lys29/Lys48-branched ubiquitin chains are considered as accelerators of PROTAC-directed targeted protein degradation, revealing a cooperative mechanism of branched ubiquitin chain assembly unique to the degradation of neo-substrates [75].

Lys11-linked ubiquitin chains may be of particular interest as a research direction for PROTAC developers, since they have proven to be critical regulators of mitotic protein degradation through the proteasome [76]. Their formation and destruction require the recruitment of a specific set of anaphase-promoting complex (APC/C), its specific chain-elongating E2, Ube2S and deubiquitinase [71, 77, 78], although it was shown that homotypic Lys11 chains did not bind to pure proteasomes or proteasome-associated ubiquitin receptors [79].

#### Monoubiquitination

It turned out that in the case of small proteins, even monoubiquitinylation is sufficient for successful interaction with the proteasome [80, 81]. Up to 50% of proteins are destroyed by the proteasome after (multi-)monoubiquitinylation [82], while modification by a single ubiquitin moiety is sufficient to target proteins with up to ∼150 amino acid residues [66], and ubiquitinylation at several lysines successfully mimic polyubiquitinylation [83]. It is possible that short proteins have less flexible domains and are less bound to the proteasome. Therefore, their binding can be stabilized by fewer modifications, shorter chains, and even a single ubiquitin fragment [66].

It is monoubiquitination that appears to be potentially the most externally controlled way to interfere with cellular mechanisms. Unlike the need to build up the “right” chains to obtain the target effect, in this case it is sufficient to carry out a single conjugation. It was shown that among ubiquitinylated proteins, the majority are monoubiquitinylated [84]. Protein monoubiquitinylation, depending on the target and site, can lead to transcriptional repression/activation, nuclear import/export, inhibition/activation of the target interaction with proteins, lipids and DNA [85–88].

As noted above, in the case of small proteins, monoubiquitinylation is sufficient for successful hydrolysis in the proteasome, but it plays a role in many processes and opens interesting prospects for practical applications. Monoubiquitinylation, including multiple, affects protein activity and protein-protein interactions; accordingly, it is involved in all cellular processes, including intracellular protein localization, endocytosis, and chromatin regulation [83, 89].

Forced monoubiquitinylation of membrane proteins is seems promising [90]. It was repeatedly shown that in the case of cytoplasmic membrane proteins, monoubiquitinylation is sufficient not only to trigger endocytosis, but also for successful endosomal sorting [91–93]. It is ubiquitin that acts as a sorting signal in the endosome membrane, where the ubiquitylated cargo is captured by the endosomal sorting complex for transport (ESCRT) machinery, which recognizes ubiquitylated cargoes and prevents their recycling and retrograde [94, 95].

#### Ub-dependent but proteasome-independent degradation of substrates

**Fig. 6:**
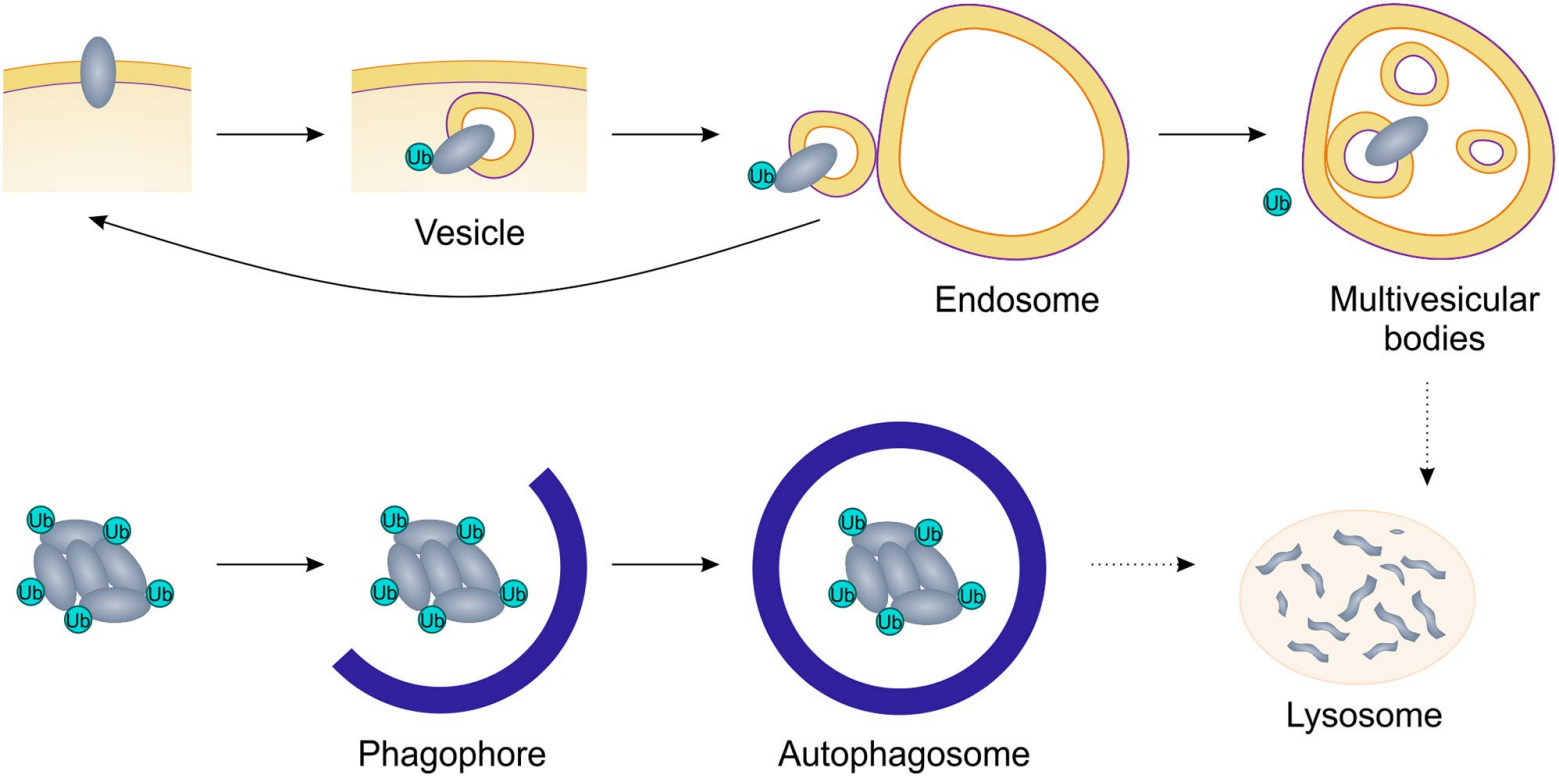
Degradation of substances through endocytosis and autophagy. Mono- or Lys63-linked polyubiquitinylation of membrane proteins acts as a signal for internalization. From early endosomes, the protein can either return to the membrane or be sorted into multivesicular bodies with subsequent degradation by the lysosome. In the case of misused or aberrantly folded proteins, ubiquitin tags initiate the macroautophagy cascade resulting in the formation of the autophagosome, the role of which is to engulf the cytosolic cargo for subsequent fusion with the lysosome

Although the PROTAC approach has significantly expanded the range of druggable proteins, it is only applicable to targets that are recognized by the proteasome, while at the same time a huge number of macromolecules, including transmembrane proteins, are not only transported, but also processed differently in the cell, e.g. through lysosomes. Ubiquitin receptors are compartmentalized along the endocytic pathway (Fig. 6) and might function as specific gating receptors for ubiquitinated cargo at different steps in the endocytic route [96]. Depending on the ubiquitin signal in the endosome membrane, proteins are sent to proteasomal degradation in the case of Lys48 ubiquitinylation, or to lysosomes in the presence of multiple monoubiquitynylation and Lys63-linked polyubiquitinylation [93]. Similar interactions with ubiquitin binding domain (UBD)-containing receptors direct mis-used or aberrantly folded proteins to the proteasome or autophagosome during selective autophagy [97]. However, the main signal for autophagy receptors appears to be the Lys63-linked ubiquitin chain [98–101]. The marking of cargo with ubiquitin is a rate-limiting step in the initiation of a macroautophagy cascade resulting in the encapsulation of the organelle in a double-membrane autophagosome [102] (Fig. 6).

Exploiting the mechanisms of ubiquitin-dependent but proteasome-independent targeting allowed the development of cargo-specific degraders, so-called AUTACs (autophagy-targeting chimera) [103]. This chimera contains a guanine tag, which is associated with subsequent K63-linked polyubiquitination of the target protein. Notably, K63-linked ubiquitinylation destines substrates for selective autophagy but is not recognized by the proteasome. The exploration of this mechanism yielded the ATTECs (AuTophagy-TEthering Compounds) technique [104]. There, the authors proposed to directly recruit LC3, a lipidated protein of autophagosome membranes, which makes it possible to provoke autophagocytosis of a wider range of targets, including those unsuitable for polyubiquitinylation [104]. The authors showed the applicability of this approach for the destruction of cellular lipid droplets (LD-ATTECs) and even mitochondria (mito-ATTECs), which opens up prospects for the specific destruction of non-proteinaceous cellular components, such as other macromolecules and organelles [105, 106]. It is worth noting that the concept of LYTAC (lysosome targeting chimera), which is close to PROTAC, is based on similar principles, where the target protein is attacked by a chimera formed by the receptor ligand that ensures the transfer of plasma membrane-associated or secreted proteins to the lysosome. For example, in [107, 108] the authors targeted the membrane-bound cation-independent mannose-6-phosphate receptor (CI-M6PR) and aasialoglycoprotein receptor (ASGPR). It can be assumed that forced activation of monoubiquitinylation or Lys63 ubiquitinylation of targets can provoke lysosomal degradation of targets.

#### Specificity of UBD and ligases

The diversity of ubiquitinylation forms provides a wide range of interactions of tagged proteins with ubiquitin-binding domains, present in many proteins (ubiquitin receptors). Most of them noncovalently bind to the hydrophobic patch around Ile44 of ubiquitin [51]. These proteins differ significantly not only in the structure of UBDs, but also in their number, which allows one to vary the spectrum of interactions and their strength. By 2012, a number of UBDs and UBD-containing proteins have been identified [109]. Importantly, UBDs can specifically recognize not only monoubiquitinylation, but also Lys48 and Lys63 chains. This broad specificity is ensured by significant differences in their spatial organization [110−114].

It was noted above that the specificity of Lys63 chains does not allow them to effectively bind to proteasomes, but they are well recognized by the corresponding UBDs. Lys63 linked ubiquitins, in turn, form a closed conformation, resulting in buried hydrophobic patch surfaces, which can still bind to UBD-containing proteins due to a constant transition between open and closed structure [115, 116]. Expanded chains are also formed when ubiquitins bind to each other in a head-to-tail manner (Met1 polyubiquitin chains, linear ubiquitin chains). Such signaling chains are critical for the NF-κB regulation and interferon induction, which prevents inflammation and regulates immune signaling [117–119]. It can be assumed that the existence of other chains (Lys6, Lys11, Lys27, Lys29, Lys33, which are not considered in this review due to their little research, also allows for highly specific interactions with individual UBDs.

Met1 ubiquitin chains can be very interesting, since they are assembled by a separate linear ubiquitin chain assembly complex (LUBAC), consisting of 2 E3 ubiquitin ligases, HOIP and HOIL-1, and the SHARPIN adapter protein. Hydrolysis of such chains can be carried out by several enzymes, among which OTULIN is specific for this type of conjugation [52, 118, 120, 121]. Similarly, Lys11 linked chains require linkage-specific enzymes: the anaphase-promoting complex (APC/C) and its specific chain-elongating E2, Ube2S; for cleavage – the Lys11 specific Cezanne [77, 122–124]. The role of such ubiquitinylation increases during cell division, when these conjugates target cell cycle regulators [122, 125].

Considering the fact that the classical PROTAC E3 ligases are Lys48 ubiquitinylating enzymes [75, 126, 127] it can be assumed that expanding the range of targeting enzymes will significantly expand the range of achievable results.

## Conclusions

Ubiquitous protein ubiquitin in the body plays the role of a multifunctional identifier, allowing cell systems to understand each other by reading a single ubiquitin code. Its use for the intended purposes opens up virtually unlimited possibilities for researchers. In addition to the induction of the classical Lys48-polyubiquitin chain, which causes proteasomal hydrolysis, rational selection of an enzyme for the specific target can provide a number of multidirectional consequences. Highly specific enzymes, which are involved exclusively in the formation of Met1 chains that lead to prevention of inflammation and regulation of immune signaling, or Lys11 chains participating in the regulation of cell division, have been identified. Forced monoubiquitinylation, which also appears to be highly selective for the specific target, will allow fine regulation of the transcriptional activity and protein localization. Monoubiquitinylation of membrane proteins, along with universal Lys63 tags, opens up opportunities for their targeted redistribution and cleavage.

The potential for E3 ubiquitin ligase recruitment is enormous, and will only expand as more information is gained about the role of ubiquitin chains with noncanonical bonds and the possibilities of using branched ubiquitin chains. The possibility of modification of nonprotein targets with ubiquitin is also interesting. It can be argued that research in these areas can provide not only scientific but also commercial results.

## Data Availability

No datasets were generated or analysed during the current study.

## Abbreviations

APC/C: Anaphase-promoting complex
ASGPR: Asialoglycoprotein receptor
AUTAC: Autophagy-targeting chimera
ATTEC: Autophagy-tethering compound
CI-M6PR: Cation-independent mannose-6-phosphate receptor
CRBN: Cereblon
LUBAC: Linear ubiquitin chain assembly complex
Lys: Lysine
LYTAC: Lysosome targeting chimera
POI: Protein of interest
SNIPER: Specific and nongenetic IAP-dependent protein eraser
SUMO: Small Ub-like modifier
Ub: Ubiquitin
UBD: Ubiquitin binding domain
UPS: Ubiquitin–proteasome system
VHL: Von Hippel–Lindau Tumor Suppressor
